# Complete genome of *Chryseobacterium* sp. strain PMSZPI isolated from the subsurface soil of a uranium ore deposit

**DOI:** 10.1128/mra.00086-25

**Published:** 2025-06-10

**Authors:** Lalitharashmi Yermunja, Celin Acharya

**Affiliations:** 1 Molecular Biology Division, Bhabha Atomic Research Centre, Mumbai, India; 2 Homi Bhabha National Institute, Mumbai, India; California State University San Marcos, San Marcos, California, USA

**Keywords:** *Chryseobacterium*, uranium ore deposit, genome analysis

## Abstract

*Chryseobacterium* sp. strain PMSZPI belongs to the phylum Bacteroidota and was isolated from the subsurface soil of a uranium ore deposit. This strain is noteworthy for its tolerance to high concentrations of uranium and other heavy metals, as well as its ability for uranium detoxification. We present here the 4.74 Mb complete genome of PMSZPI to gain insights into its adaptation and survival strategies in soil enriched with uranium and other heavy metals.

## ANNOUNCEMENT

*Chryseobacterium* members belonging to phylum Bacteroidota are aerobic, chemoorganotrophic, gram-negative, and non-spore-forming rods (1). Members of this genus are prevalent in the environment, including coal mines (2), volcanic sites (3), wastewater (4, 5), nuclear-spent fuel pools (6, 7), and oil-contaminated sites (8, 9). In our previous study, we reported the draft genome (GenBank accession no. PIZV01) of uranium-tolerant strain, *Chryseobacterium* sp. strain PMSZPI (10). The strain PMSZPI exhibited remarkable tolerance to heavy metals (10) and uranium biomineralization capability (11). In this study, the complete genome sequence of *Chryseobacterium* sp. PMSZPI was generated through whole genome *de novo* hybrid sequencing.

**Fig 1:**
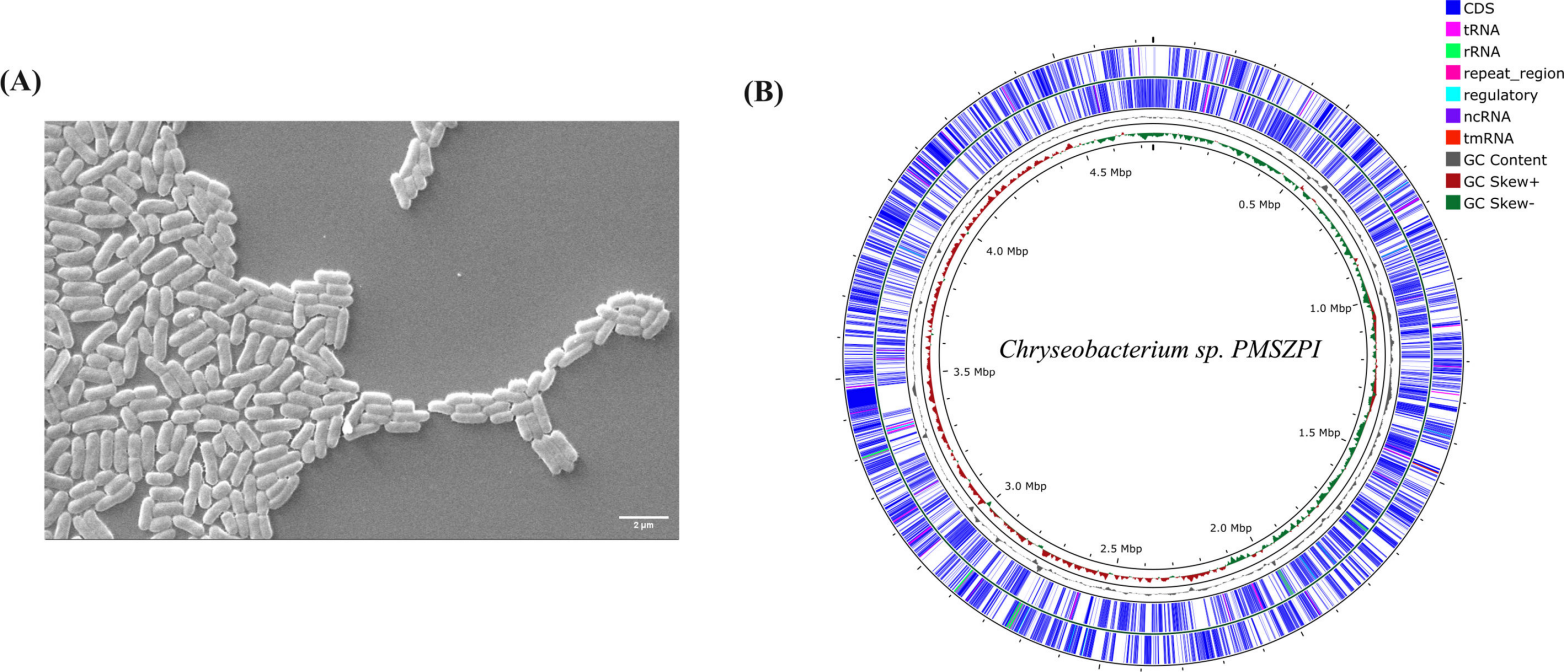
*Chryseobacterium* sp. PMSZPI and its genome organization. (**A**) A representative scanning electron micrograph of PMSZPI cells demonstrating rod-shaped morphology. (**B**) Genome architecture of *Chryseobacterium* sp. PMSZPI. The circular map was generated using Proksee version 1.0.0a6 (15).

The strain was isolated from subsurface soil of the Domiasiat Phudsyngkai uranium (U) mining site (GPS N25°19.140′: E91°12.705) in Meghalaya, India (Fig. 1A). Soil samples were collected at a depth of 15–30  cm from the surface in sterile plastic vials and inoculated in low phosphate medium (LPM) at pH 7.5 amended with 1 mM U(VI) added as uranyl nitrate salt (Sigma) and incubated at 30°C for 48 h to enrich U-tolerant organisms (12). The resulting enriched cultures were subsequently streaked onto LPM agar plates supplemented with 1 mM U(VI) to obtain isolated colonies. The pure culture of PMSZPI thus attained was identified using 16S rRNA sequencing (12). The genomic DNA was isolated from PMSZPI cells using a DNeasy Blood and Tissue kit (Qiagen 69506). For PacBio Sequel II sequencing, purified gDNA was sheared by Megaraptor 3, analyzed for DNA size using an Agilent FEMTO pulse analyzer (Agilent Technologies, CA, USA), and purified using SMRT Bell cleanup beads and AMPure PB beads (Pacific Biosciences, CA, USA). A SMRTBell pool library size of 8,612 bp was constructed using the SMRTbell Express Template Preparation Kit 3.0 (Pacific Biosciences). The library was then loaded onto SMRTcell containing 8M ZMW and was sequenced in the PacBio Sequel II system in CCS/HiFi mode, generating 18,134,780,730 polymerase read bases. The generated PacBio subreads were converted, and a total of 138,551 CCS HiFi reads with an average length of 8,196 bp and an *N*_50_ of 9,267 bp were obtained. For Illumina sequencing, gDNA library was prepared using KAPA HyperPlus Kit (cat no-KR1145-v8.21) with an average size of 315 bp and sequenced in Illumina NovaSeq 6000 using S4 flow cell. Subsequently, Illumina paired-end sequencing generated 41,298,920 reads with an average read length of 151 bp. Quality control for sequenced data was performed using FastQC (version 0.12.1) (13), and the reads were trimmed for low-quality bases (<Q20) and adapters using Fastp (version 0.23.4) (14).

The obtained CCS HiFi reads were *de novo* assembled by Trycycler (version 0.5.5) using default parameters (16). This generated circular consensus genome was polished with Illumina reads using NextPolish (version 1.4.1) (17). The assembly quality was assessed using BUSCO (version 5.1.3) (18). The assembly assessment revealed that the maximum BUSCOs in the sample were complete and present as single-copy genes, with a few duplicated or fragmented genes. The assembled genome of PMSZPI contained a single contig of 4.7 Mb with 225× genome coverage and 35% GC content (Table 1). Furthermore, the genome was annotated by the NCBI Prokaryotic Genome Annotation Pipeline (PGAP 2024-07-18.build7555) (19). *Chryseobacterium* sp. C4a (GenBank accession no. WXVU00000000) was found to be the closest strain to the assembled *Chryseobacterium* sp. PMSZPI genome by both phylogenetic and BLASTp (version 2.14.1) analysis. The annotated circular genome map with its features was visualized using Proksee version 1.0.0a6 (Fig. 1B) (15).

**TABLE 1:** Assembly and genome features of *Chryseobacterium* sp. PMSZPI

Description	Values
Genome size	4.7 Mb
Number of contigs	1
Contig *N*_50_	4.7 Mb
Contig *L*_50_	1
GC percent	35
Assembly level	Chromosome
CDS	3,977
Gene	4,099
tRNAs	89
rRNAs	18
ncRNAs	3
Pseudogenes	12

## Data Availability

The raw sequencing data are deposited in Sequence Read Archive (SRA) database (https://www.ncbi.nlm.nih.gov/sra) under accession numbers SRX26470921 and SRX26470920. The genome assembly is deposited in the GenBank database (https://www.ncbi.nlm.nih.gov/genbank) under accession number CP178384.
